# Causal Link of Human Papillomavirus in Barrett Esophagus and Adenocarcinoma: Are We There Yet?

**DOI:** 10.3390/cancers15030873

**Published:** 2023-01-31

**Authors:** Shanmugarajah Rajendra, Prateek Sharma

**Affiliations:** 1 Gastro-Intestinal Viral Oncology Group, Ingham Institute for Applied Medical Research, Liverpool, Sydney, NSW 2170, Australia; 2 South Western Sydney Clinical School, University of New South Wales, Kensington, Sydney, NSW 2052, Australia; 3 Department of Gastroenterology & Hepatology, Bankstown-Lidcombe Hospital, South-Western Sydney Local Health Network, Bankstown, Sydney, NSW 2200, Australia; 4 Division of Gastroenterology and Hepatology, Veterans Affairs Medical Center, Kansas City, MO 64128, USA; 5 School of Medicine, University of Kansas, Kansas City, MO 66160, USA

**Keywords:** human papillomavirus, Barrett’s esophagus, cancer, infection, esophageal adenocarcinoma

## Abstract

**Simple Summary:**

Esophageal cancer is not an uncommon malignancy in the world with a relatively high mortality. There has been a dramatic upward trajectory (approximately 400%) in the number of patients diagnosed with the glandular form of esophageal cancer (adenocarcinoma) since the 1970’s. It has been thought that this condition is related to chronic heartburn causing Barrett’s esophagus (a pre-cancerous condition), smoking and obesity. Nevertheless, this excess number of esophageal cancers is not entirely explained by these known risk factors despite improved screening, detection and treatment strategies. The discovery of high-risk type human papillomavirus (with an increased cancer potential) being strongly associated with this glandular form of tumor of the esophagus in a subset of patients (approximately 25%) may partially explain this anomaly. This discovery will aid improved detection rates of those with a greater risk of progressing to malignancy and modified treatment strategies including vaccination in the hope of reduced mortality.

**Abstract:**

Esophageal cancer is a relatively common malignancy worldwide with a high mortality (5-year survival of <15%). Despite screening, surveillance, improved imaging and treatment, the exponential rise in OAC continues. The strongest risk factors for OAC are chronic heartburn and metaplastic transformation of the lower third of the esophagus (Barrett’s esophagus). The risk profile includes Caucasian race, male gender older age, obesity and smoking. Although the tumor risk in BO has been progressively revised downwards, the exponential rise in OAC remains unchecked. This paradox points to an unidentified missing link. Relatively recently, we provided the world’s initial data for a strong association of biologically relevant hr-HPV with BD and OAC. Since then, systematic reviews and meta-analysis have documented HPV DNA prevalence rates in OAC of between 13 to 35%. In this review, we provide some evidence for a probable causal relationship between hr-HPV and OAC. This is challenging given the multifactorial etiology and long latency. Increasingly, high-risk HPV (hr-HPV) is regarded as a risk factor for OAC. This discovery will aid identification of a sub-group of high-risk progressors to esophageal cancer by surveillance and the development of effective preventive strategies including vaccination.

## 1. Introduction

Fifteen to twenty percent of all human malignancies are caused by bacterial or viral pathogens [1,2]. Helicobacter pylori, high-risk human papillomavirus (hr-HPV), hepatitis B virus and hepatitis C are responsible for more than ninety percent of pathogen related cancers [3,4]. Most of these oncogenic pathogens are modifiable risk factors with either effective treatments and/or prevention strategies in place [1].

Epidemiological and basic science studies have demonstrated that hr-HPV causes cervical, anogenital, and some oropharyngeal cancers [5,6] This virus has been detected in the vast majority of squamous cervical tumor specimens (90–100%) as compared to a minority of controls (20%) [7]. In addition, the HPV viral genome has been identified in a significant majority of cervical dysplastic lesions [8]. HPVs were previously thought to preferentially infect squamous epithelia only. More recently, studies have established a consistent and strong association between hr-HPV and adenocarcinoma of the cervix. Eighty-five percent of this heterogeneous group of malignancies are HPV associated [9,10]. In this respect, HPV exhibits an affinity for glandular tissue as well as squamous epithelium.

## 2. Literature Search and Study Selection

This is a narrative review and articles were evaluated in Pubmed, MEDLINE, Scopus and Google Scholar between 1960 and 2022 except for one seminal publication by Koch (English translation from German) published in 1881. This article was identified in the course of review of references of the evaluated publications to locate further relevant research.

We searched publications relevant to the title of the review using the following search terms: human papillomavirus, HPV, viruses, viral pathogens, oncogenic viruses, HPV integration, HPV associated cancers, esophageal adenocarcinoma, esophageal cancer, esophageal tumor, esophagus, Barrett’s esophagus, Barrett’s metaplasia, Barrett’s dysplasia and epidemiology.

Publications deemed suitable for inclusion in this review were (i) all in English or translated into English, (ii) full-text articles, (iii) related to the above search terms. This resulted in a total of 7042 articles. Studies pertaining only to squamous cell carcinoma of the esophagus or gastro-esophageal junction tumors or cardia malignancies without separately including OAC were excluded. In addition, duplicate and irrelevant publications were also excluded. Ultimately, a total of 108 articles were included in the review.

## 3. HPV as a Common Denominator in Esophageal Adenocarcinoma and Cervical Cancer

One of the authors has previously hypothesized (SR) that HPV could be involved in the pathogenesis of esophageal adenocarcinoma just as in cervical cancer. It is based on the premise of similar immunology and genetics and a not dissimilar transformation zone that could be a common predilection site for the virus [11]. Papillomatous lesions have been identified in the esophagus which throws up the possibility of a viral pathogen (biological plausibility) [12]. In fact, up to 10% of esophageal squamous cell papillomas have detectable HPV [13]. Other viruses, namely Herpes simplex virus (HSV), cytomegalovirus (CMV) and Epstein-Barr virus (EBV) have all been detected in the esophagus and, in a few publications, have even been associated with cancer [14]. In the animal world, cattle suffer with upper gastro-intestinal tract papillomas and carcinomas which have been linked to the bovine papillomavirus (BPV 4) and bracken fern (co-factor) (animal model) [15]. BPV, although species-specific, belongs to the Papillomavirus genus. Most BPV infections in cattle regress but when the animals feed on bracken fern the papillomatosis persists with cancer formation both of epithelial and mesenchymal origin [16]. Moreover, the transforming activity of BPV preparations in bovine cells has been demonstrated [17].

We have proceeded to undertake studies to confirm or disprove the above hypothesis and will discuss these in detail as well as investigations undertaken by others, showing both positive and negative associations. More importantly, we shall frame the data in a manner consistent with the title of this review.

## 4. Establishing Causality

Establishing that a virus causes cancer is a tedious undertaking with a high barrier for acceptance by the scientific community [2]. Attributing causality to microbes in human cancers has to take into account (a) the long latency between infection and cancer development, (b) that only a small number of infected individuals develop malignancies, (c) that persistent chronic infection is a key precursor event, (d) that host and environmental co-factors are a necessary pre-requisite and (e) possible strain-dependent variation in oncogenicity.

The existing model in the development of esophageal glandular carcinogenesis clearly only refers to chronic heartburn/reflux and Barrett’s esophagus (BO). Chronic, frequent and severe GORD symptoms are strongly associated with OAC (odds ratio (OR) of 43.5) [18]. Barrett’s metaplasia is considered a pre-malignant condition that can lead to OAC. The malignancy potential ranges between 0.12 and 0.13% per annum [19,20]. In the past, the cancer risk was thought to be approximately eight times higher at 1.0% per year (one case per 125 patient-years). This estimated risk has been reduced over time to as low as one case per 200 to 300 patient years [21,22].

Herein lies the dichotomy. OAC has been growing at an exponential rate in the Western world, i.e., by 600% since the 1970’s [23], although recently, the rate of rise has stabilized in the US and Sweden [24,25]. This exponential rise of OAC has occurred against a background of a reduction in the cancer risk linked with BO. It has been postulated that to “explain a rise of esophageal adenocarcinoma of this magnitude, the prevalence of a strong risk factor must also rise exponentially” [23]. The finding of a strong association of biologically relevant high-risk human papillomavirus (hr-HPV) with Barrett’s dysplasia (BD) and cancer [26] may potentially explain this anomaly. The data on head and neck tumors, another type of virally linked cancer [27,28,29], provides further credence to this statement. The prevalence of HPV in oropharyngeal cancers (OPC) increased more than four-fold in 20 years from 16% in the 1980’s to 73% in the 2000s (incidence: 0.8 cases/100,000 subjects to 2.6/100,000) [27]. Is there a common denominator here in regard to hr-HPV and OAC and oropharyngeal malignancies?

The usual criteria for establishing causality are the consistency of association at the epidemiological and/or molecular level and the ability of the pathogen to cause tumors in animal models or its transforming ability in cell culture [30].

Assigning causality in medical research usually involves adhering to Koch’s postulates. These state that a pathogen be found in all hosts with disease but not in those without but with the exception of asymptomatic carriers. Furthermore, the microbe must be able to be identified and shown to cause disease in a healthy host [31].

The Hill criteria examine (a) the robustness of association, (b) consistency, (c) temporality, (d) biological gradient, (e) biological plausibility, (f) biological coherence, (g) specificity, (h) experimental evidence and (i) analogy [32]. Lipkin has amalgamated both Koch’s postulates and Hill’s criteria and graded causality as possible, probable, or confirmed. A “possible relationship” has been defined as evidence of exposure to a microorganism, the detection of genetic sequences/proteins, visualization by imaging, or demonstration of an adaptive immune response to a pathogen. A “probable relationship” is one where there is a template for a comparable disease caused by a similar pathogen in a related host (analogy), and the biological gradient is greatest at the site of pathology. This may include presence of pathogen genetic material or protein particles in or adjacent to diseased host cells, or IgM antibodies against the microbe indicating recent exposure. A “confirmed relationship” requires adherence to Koch’s postulates or establishing that the disease can be ameliorated or prevented by pathogen specific drugs, antibodies, or vaccines [33].

## 5. Consistency across Studies and Biological Gradient (Probable Relationship)

A systematic review reported a pooled HPV prevalence rate of 35% (95%CI, 13.2–65.7%) from 5 studies which totaled 174 patients (predominantly Caucasians) with OAC [34]. Another review of 19 studies found the prevalence of HPV in OAC was 13% (95% CI: 2–29%) (Table 1). The investigators opined that the low detection rate may have been due to small sample sizes and unreliable assays [35]. The shortcomings of the meta-analysis included lack of data to analyze genotypes, primer types and geographical variations, and paucity of studies which compared HPV prevalence in subjects with BO and OAC or those with normal esophageal epithelium. Some of these studies used unsatisfactory classification of tissue histology. Many used a less reliable non-nested PCR method, failed to obtain biopsies from the transformation zone (squamo-columnar junction rather than the gastro-esophageal junction) and most either lacked proper controls or were devoid of them altogether [36,37,38,39]. The low viral load in esophageal epithelium further exacerbates the problem [26,40]. Very recently, the largest meta-analysis to date on the association between the virus and both sub-types of esophageal cancer, i.e squamous and adenocarcinoma, involving 13,401 subjects (33 studies of which 19 were case-control, 11 cohort/observational and 3 cross-sectional) revealed that hr-HPV 16 and 18 are strong co-factors in the etiopathogenesis of esophageal cancer [41].

## 6. Strength of Association and Biological Gradient (Probable Relationship)

In 2013, we published for the very first time 2 papers in the American Journal of Gastroenterology demonstrating a strong link between transcriptionally active high risk genotypes 16 (predominantly) and 18 with Barrett’s dysplasia and OAC [26,40]. HPV DNA detection was undertaken by nested polymerase chain rection (PCR) and viral oncogene activity (indicative of viral transcription) estimated by E6/7 oncogene messenger ribonucleic acid (mRNA) expression (gold standard) and p16INK4A immunohistochemistry in esophageal tissue of patients with dysplastic and non-dysplastic (metaplastic) Barrett’s, and OAC, as well as controls. Both junctional and lesional biopsies were obtained for analysis.

We found that 81/261 (31%) patients had detectable HPV DNA in the esophagus. In both controls and BO, the virus was more likely to be located at the transformation (transitional) zone. When compared with controls (18.0%), HPV positivity was significantly more common in BD (68.6%) and OAC (66.7%), but not in BO (22.1%) [26].

Of the viral positive patients, approximately 93% were high-risk (HR) HPV, i.e., types 16 and 18. In addition, p16INK4A expression was greater in BD (44.1%) and OAC (44.4%) and significantly reduced in BO patients (10.6%).

In the 66 viral positive subjects, tested for E6/E7 oncoproteins, none of the controls (n = 16) or those with Barrett’s metaplasia (n = 13) were positive. In contrast, 9/22 (40.9%) BD and 9/15 (60%) OAC patients had detectable E6/E7mRNA (*p* < 0.001). When all 3 markers were positive, (HPV DNA, p16INK4A, and E6/E7 mRNA), a strong association with disease severity was noted as compared with none detected (fulfills criteria for probable relationship). This strength of association is as strong as chronic GORD for OAC [18].

Viral infections in the esophagus have a predilection for the transformation zone. In both dysplastic/OAC subjected to endoscopic mucosal resection, high-risk genotypes 16 and 18 were concentrated at the transition zone (SCJ) compared to the lesion (biological gradient, plausibility, and specificity) [11,42,43,44]. Transforming HPV infections in varying epithelial sites (ano-genital including cervix and oropharynx) cause cancers mainly at the transition zone (analogy). In the cervix, this is the anatomical area where metaplastic squamous cells are detected in otherwise columnar-lined endocervical glands [45,46,47]. Intriguingly, studies have revealed that Barrett’s metaplastic tissue can originate from a distinct category of embryonic cells located at the junction of the transition zone. Moreover, in a genetically engineered animal (mouse) model, Barrett’s metaplasia and dysplasia was generated from the SCJ (biological mechanism and experiment) [48,49].

## 7. Analogy and Biological Gradient (Probable Relationship)

The higher the viral load the greater the risk of developing cervical cancer and its precursor lesion, cervical intraepithelial neoplasia (CIN) which is the archetypal HPV driven disease. Nevertheless, this is not necessarily the case in other HPV associated lesions, where viral load does not correspond to disease severity [50,51,52]. Incorporation of the viral DNA into the host genome is an important step in malignant conversion. Generally, frequency of hr-HPV DNA integration corresponds to increasing severity of cervical lesions [53,54]. Integration leads to disruption of the HPV *E2* gene, and thus unchecked over-expression of viral E6 and E7 oncogenes [55].

**Figure 1:**
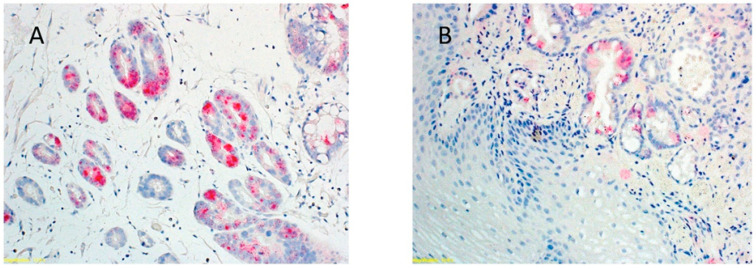
E6/E7 oncogene analysis of hr-HPV 16 and 18 using a novel in-situ hybridization detection technique (RNAScope) in OAC (**A**) and Barrett’s dysplastic (**B**) tissue. Positive staining is diffusely present in the nuclei and cytoplasm as punctate/granular appearance. Oncogene activity is only evident in columnar cells. No staining seen in adjacent squamous tissue (**B**).

Work undertaken in our laboratory has revealed that a greater abundance of virus as per viral load measurement and greater integration of the pathogen in human esophageal tissue involving the HPV genotypes 16 or 18 was significantly associated with disease severity. This severity increased in ascending order as per the Barrett’s metaplasia-dysplasia-adenocarcinoma sequence [40]. Patients in the benign end of the spectrum were more likely to be infected with the HPV-18 genotype. This was in contrast to the cohort with more severe disease (BD and OAC subjects) in whom the predominant HPV genotype was 16 [40]. HPV DNA in situ-hybridization (ISH) and E6/E7 mRNA expression was confined to columnar lined esophageal lesions only. It was quite intriguing to find that the squamous epithelium was totally spared of any positive detection signals (Figure 1). This clearly indicates that HPV has a tropism for esophageal glandular tissue. An analogous model is the affinity of HPV18 for columnar cells of the endocervical canal [56]. In the latter disease, the columnar cells are not suited for HPV reproduction as they are unfavorable to the establishment of a differentiation gradient, contrary to squamous epithelia. What is baffling is that despite the absence of a productive viral infection, hr-HPV can still cause cancer of the endocervical canal [57].

## 8. Analogy and Experimentation [Probable Relationship]

In the cervix, continual hr-HPV infection is associated with CIN and frank carcinoma [58,59]. Prolonged uninterrupted hr-HPV infection is a predictor of failed treatment (loop electrosurgical excision (LEEP)) of CIN [60]. In an analogous fashion, we investigated whether HPV infection and p53 over-expression (as determined by IHC and next generation sequencing) was abolished in virally infected patients after endoscopic treatment for BD and early esophageal adenocarcinoma (intramucosal). In this prospective study which involved 40 patients, it was found that persistent biologically active hr-HPV infection (types 16 and 18) and p53 over-expression were mutually exclusively associated with failed endoscopic ablation of dysplastic BO/OAC [61]. HPV positive BD/intra-mucosal OAC had largely absent p53 mutations and next generation sequencing confirmed wild type TP53 in these lesions. This is a hallmark of HPV driven cancers [62,63].

The failure to detect p53 in BD may be due to silencing mutations of the said gene which results in reduced or absent expression [64] It may also be the case that this negative staining for this tumor suppressor gene is due to the E6 degradation of p53 [65]. It is tempting to postulate that there maybe 2 or more carcinogenic pathways (viral and non-viral) involved in esophageal carcinogenesis, much akin to head and neck tumors [65,66,67], one which is HPV driven, i.e., mostly absent p53 mutations and the other, p53 mediated. Validation of our findings by others is paramount prior to contemplating translation from bench to bedside.

## 9. Comparative Genomic Analysis Reveals Distinct Differences between HPV-Positive and HPV Negative OAC (Probable Relationship)

HPV+ tumors represent a unique subgroup of cancer with distinct prognosis and genetic aberrations. HPVs exert their oncogenic role via the viral oncoproteins E6 and E7. These oncoproteins target tumor suppressor signaling pathways which are critical for modulation of cellular growth. E6 oncoprotein induces degradation of p53 [68], whereas E7 binds and breaks down the pRB retinoblastoma tumor suppressor protein [69]. Due to feedback loops from pRb loss, up-regulation of *p16INK4a* occurs in viral positive tumors which serves as a proxy marker for the presence of the active HPV oncoprotein, E7. Compared with HPV-negative HNSCC (which is caused by life-style habits, e.g., smoking and excess alcohol intake), HPV-positive cancers have less genome-wide DNA copy number alterations, reduced genome-wide hypomethylation, quite infrequent or absent *TP53* mutations, and reduced expression of EGFR [70,71]. In another study involving targeted next-generation sequencing of HPV+ and HPV− head and neck squamous cell carcinoma (HNSCC), TP53 mutations were detected in all (100%) of HPV negative cases. In contrast, only 1/20 (5%) had TP53 mutations in the HPV+ group [68].

A pilot study involving whole exome sequencing of 4 HPV associated OAC and 8 non-HPV OAC revealed that viral-positive OAC subjects had half the number of somatic (non-silent) mutations compared with virus-negative esophageal cancer patients [72]. TP53 mutations were non-existent in the HPV-positive OAC group whereas half of the HPV-negative OAC patients exhibited TP53 aberrations. There were less than a third of cancer driver genes in the HPV+ group as compared with the viral negative cohort. The integrated form of HPV16 was identified in a few discordant pairs [72]. Exomes make a tiny fraction of the human genome, and it is speculated that this viral integration is a rather unpredictable process [73]. Hence, larger sized studies examining the whole genome are necessary to further confirm these results. For example, protein expression studies which may explain function of these mutated gene are warranted for validation purposes. They may also shed light on esophageal carcinogenesis. Unfortunately, no comparable studies by other investigators have been reported on genomic analysis of HPV+ versus HPV-OAC.

A prospective cross-sectional study investigating the prevalence of biologically active HPV virus and related protein markers in patients representing the Barrett’s metaplastic, dysplastic and adenocarcinoma sequence has shed some light on the molecular mechanisms involved in a subgroup of virally associated esophageal lesions. In this investigation, 56/218 patients were HPV DNA positive (HPV16 (n = 42), genotype18 (n = 13), genotype 6 (n = 1)). Biologically active HPV (DNA^+^/RNA^+^) was only found in the dysplastic and adenocarcinoma group (n = 21). The majority of HPV DNA^+^/RNA^+^ BD/OAC exhibited over-expression of p16, with reduced or absent staining of pRb and p53_._ This finding was significantly different to controls. Reduced or absent expression of p53 had the strongest association with DNA^+^/RNA^+^ esophageal lesions (OR = 23.5, *p* = 0.0029). Sequencing confirmed the overwhelming majority of p53_low_ specimens exhibited wild-type status. pRb_low_/p53_low_ provided the optimal strength of association for DNA^+^/RNA^+^ BD/OAC. Hence, this study concluded that active HPV involvement in the pathogenesis of a subset of BD/OAC is characterized by wild-type p53 and aberrations of the retinoblastoma protein pathway [74].

## 10. Experimentation (Evidence for Possible Relationship)

Modern techniques facilitate concurrent analysis of antibodies against a broad range of viruses including HPV. Antibodies to HPV6/11/16/18/31/33/45/52/58 and E6/E7/E1/E2 as well as L1 antigens were analyzed using multiplex technology in 438 patients representing the GORD spectrum disease. These patients were categorized as hospital/reflux controls and cases were defined as Barrett’s metaplasia (BM), BD and intramucosal EAC. Seropositivity for individual HPV proteins was approximately 10% in both cases and controls. More specifically, there was no difference in antibody seroprevalence to any HPV antigen/antibody combination between cases and controls. Among HPV16 DNA+ BD/OAC subjects, antibodies to HPV 16 E7 oncoprotein were significantly more prevalent (11.5%) as compared to controls (1.5%). In HPV18 DNA+ cases, antibodies to HPV18 E1 protein were present in 50% of cases versus 1.5% of controls (*p* = 0.0002) [75]. Antibodies to E6 and E7 cancer proteins are considered markers of HPV-driven cancer, namely head and neck and cervical malignancies [76,77,78,79,80]. L1 antibodies denote exposure, or cumulative infection of HPV [81,82]. Antibodies against E1 and E2 for HPV 16 have been shown to be additional cancer biomarkers in oropharyngeal cancer (OPC) [83]. Larger studies encompassing patients with more severe disease have been undertaken with mixed results. Lagergren et al. tested IgG against HPV 16/18 capsids in 173 OAC (Siewert Class I and II), 121 OSCC and 302 controls and their only significant (unexpected) finding was a negative association between HPV18 and OAC. The authors related this anomaly to possible bias [84]. Site-specific correlation, i.e., testing the esophageal lesion for HPV DNA status and correlating it with serological analysis was not undertaken. Their results differed from 3 prior published positive studies [85,86,87]. In the latter 2 studies, HPV16 seropositivity was associated with a 6 times excess risk of esophageal malignancy (mainly squamous carcinoma). Again, the downside to these studies was that site-specific serological correlation was not performed [86,87].

## 11. HPV and Survival in OAC (Probable Relationship)

HPV confers a favorable prognosis in Barrett’s high-grade dysplasia and EAC suggesting a distinct tumor biology as compared with viral negative esophageal lesions [72,88]. In 142 patients with Barrett’s high-grade dysplasia (HGD) and EAC (37 HPV-positive and 105 HPV-negative), there was a superior disease-free survival (DFS) in the viral-positive group (40.3 vs. 24.1 months; *p* = 0.003) and an improved overall survival (OS) [43.7 vs. 29.8 months; *p* = 0.009].

Similarly, an improved disease-free survival (DFS) was demonstrated for HPV, biologically active virus, E6/E7 mRNA and high p16INK4A expression associated Barrett’s HGD and OAC. This was due to a reduction in distant metastasis and probably improved loco-regional control in virally infected patients. These findings mirror the data in head and neck cancers (HNSCC) with improved survival and reduced loco-regional recurrence in HPV+ lesions tumors versus viral negative head and neck malignancies. The exception was findings of no difference in distant metastases due to underpowered studies [89,90,91]. In OSCC, p16+ patients had superior 5-year OS rates and progression-free survival (PFS) as compared to those lacking this biomarker [91]. Likewise, another study investigating p16 status in OSCC patients subjected to neo-adjuvant chemotherapy found that tumors positive for this biomarker had superior complete remission rates than those without. [92]. Another study by Wang et al. concurred with the above findings. They reported that patients with HPV-16 positive advanced OSCC, had a significantly improved survival and response to chemoradiotherapy than those with viral negative esophageal tumors [93].

Conversely, Furihata et al. found that HPV positivity and the presence of p53 conferred a poorer prognosis in OSCC patients than those without the virus or absent p53 [94].

In a related study, da Costa et al. found HPV, p16 and p53 had no bearing on prognosis in OSCC. [95]. Others have also shown no survival benefit between HPV positive versus viral negative OSCC [96,97,98].

In another study, investigating some of the HPV associated biomarkers, namely, retinoblastoma protein (pRb), cyclin D1 (CD1), mini-chromosome maintenance protein (MCM2) and Ki-67 in relation to survival in patients with HGD/OAC found that only low cyclin D1 levels were associated with a favorable prognosis, specifically for OS [99]. Overexpression of cyclin D1 in OAC has been well documented [99,100]. Nevertheless, when pRb, CD1, MCM2, and Ki-67 were stratified by human papillomavirus status there was a survival benefit in esophageal tumours [101]. The above findings suggest the enticing possibility of personalization of therapy predicated on viral status. We caution that these findings have to be corroborated by other investigators in larger cohorts, preferably with more severe disease. Only then could one begin to contemplate treatment de-escalation and the potential benefit of reduced toxicity without adversely affecting survival.

## 12. HPV and Esophageal Cancer Cell Lines

Hr-HPV genotype 18 has been isolated from esophageal squamous cell carcinoma cell lines and the authors identified the integration site to a part of chromosome 8. This may represent a possible molecular mechanism for viral oncogenesis in esophageal malignancy [102]. Boon et al. analyzed the transcript expression profiles and functions of E6 and E7 in order to decipher the function of HPV18 in esophageal cancer cell lines. They found disruption of E2, and expression of E6, E7, E1 and L1 transcripts just as in HPV driven cervical cancer. A point of differentiation was that E7 preferentially targeted p130 in the two esophageal cancer cell lines, instead of pRb as in cervical cancer [103].

## 13. Conclusions

Further replication of the studies alluded to above is paramount in establishing a causal relationship between HPV and a subset of Barrett’s dysplasia and OAC. Thus far, the International Agency on Research on Cancer (IARC) concluded in 2012 that there was inadequate epidemiological evidence for an association between esophageal cancer (squamous cell carcinoma) and HPV based on case series and case-control studies [3]. Since then, at least 3 systematic reviews have been published demonstrating a positive association between HPV and OAC together with a few studies addressing molecular and genomic signatures characteristic of these virally driven BD and OAC [26,34,35,40]. Unfortunately, the Advisory Group recommendations on priorities for the IARC Monographs during 2020–2024 place HPV as a low priority for evaluation, though virologists worldwide will be making submissions for upgrading this assessment [104].

In the interim, we believe there is accumulating evidence for a probable relationship for hr-HPV in the etiology of a sub-group of OAC based on the data provided above. In fact, hr-HPV continues to gain traction as a risk factor for esophageal adenocarcinoma on the basis of more recent investigations, meta-analyses and reviews. [41,105,106,107,108]. The largest meta-analysis conducted so far on both squamous and adenocarcinoma of the esophagus involving over 13,000 subjects has concluded that HPV 16 and 18 are both probable strong risk factors in the pathogenesis of the disease [41]. Critical factors in the understanding of HPV oncogenesis in esophageal adenocarcinoma remain unresolved. Both genomic and proteomic studies will provide mechanistic insights for OAC with and without a viral etiology. Furthermore, the confirmation of a distinct molecular signature via sequencing and proteomic analysis characteristic of HPV positive esophageal malignancy would greatly aid risk stratification and improved/modified treatment protocols for patients, with the potential of reduced toxicity. The identification of a sub-group of high-risk progressors to esophageal cancer by surveillance and the development of effective preventive strategies including vaccination would have a great impact on public health. The potential histopathological reversal (regression) of a subset of patients with Barrett’s dysplasia via HPV therapeutic vaccination is the holy grail we aspire to in the near future.

Part of this data was presented at an invited lecture of an identical title by SR at the Asia Pacific Digestive Week, Kuala Lumpur, 19–22 August 2021.

## Figures and Tables

**Table 1:** Studies included in systematic review by Kunzman et al. (with permission) [35].

Geographic Region	No. of Samples Tested	OAC	BO	No. of HPV-Positive Samples	HPV Prevalence (%)	No. of Reports
USA	231	16	23	39	16.8	8
UK	141	68	73	9	6.38	5
Germany	211	8	0	8	3.79	4
Australia	340	228	112	22	6.4	2
Italy	23	23	0	3	13	2
Turkey	29	29	0	6	20.7	2
Mexico	45	17	28	42	93.3	1
Netherlands	48	48	0	11	22.9	1
Iran	4	0	4	1	25	1
Sweden	27	27	0	5	18.5	1
South Africa	1	1	0	0	0	1
China	57	0	0	0	0	1
Korea	3	3	0	0	0	1
France	40	40	0	0	0	1
India	5	5	0	0	0	1

OAC, esophageal adenocarcinoma, BO, Barrett’s esophagus.
